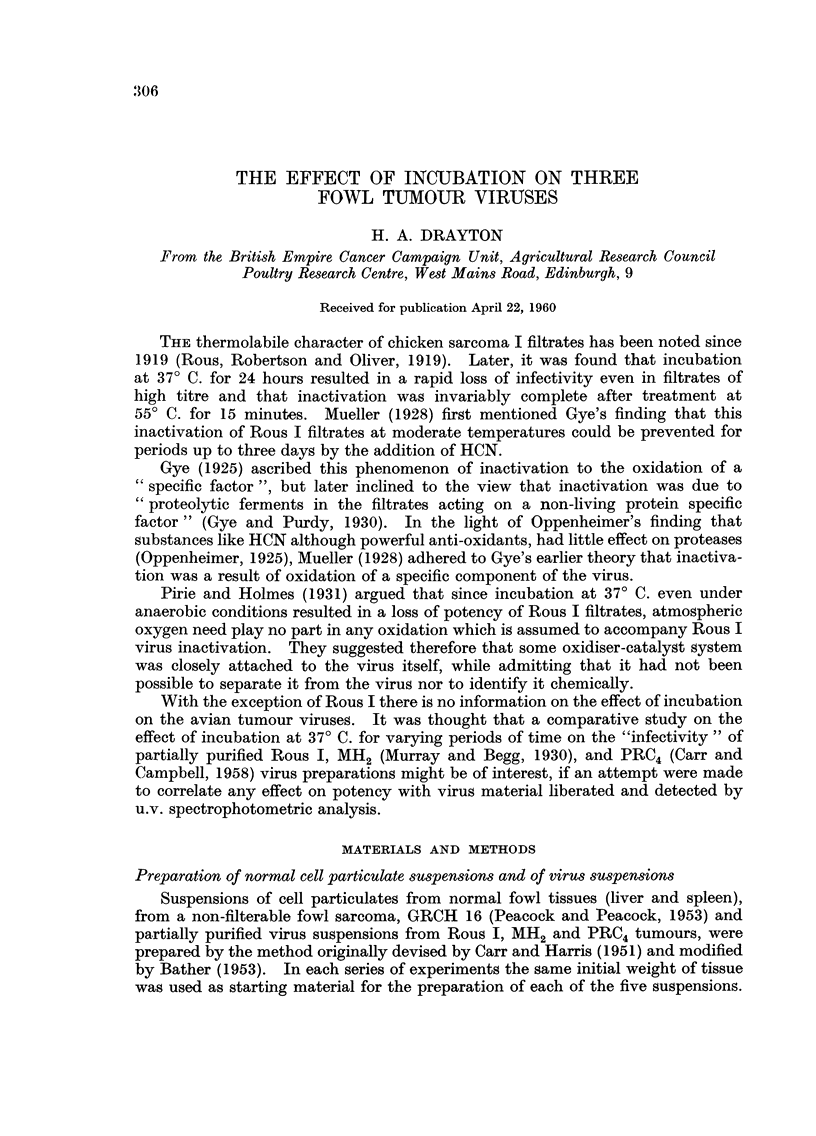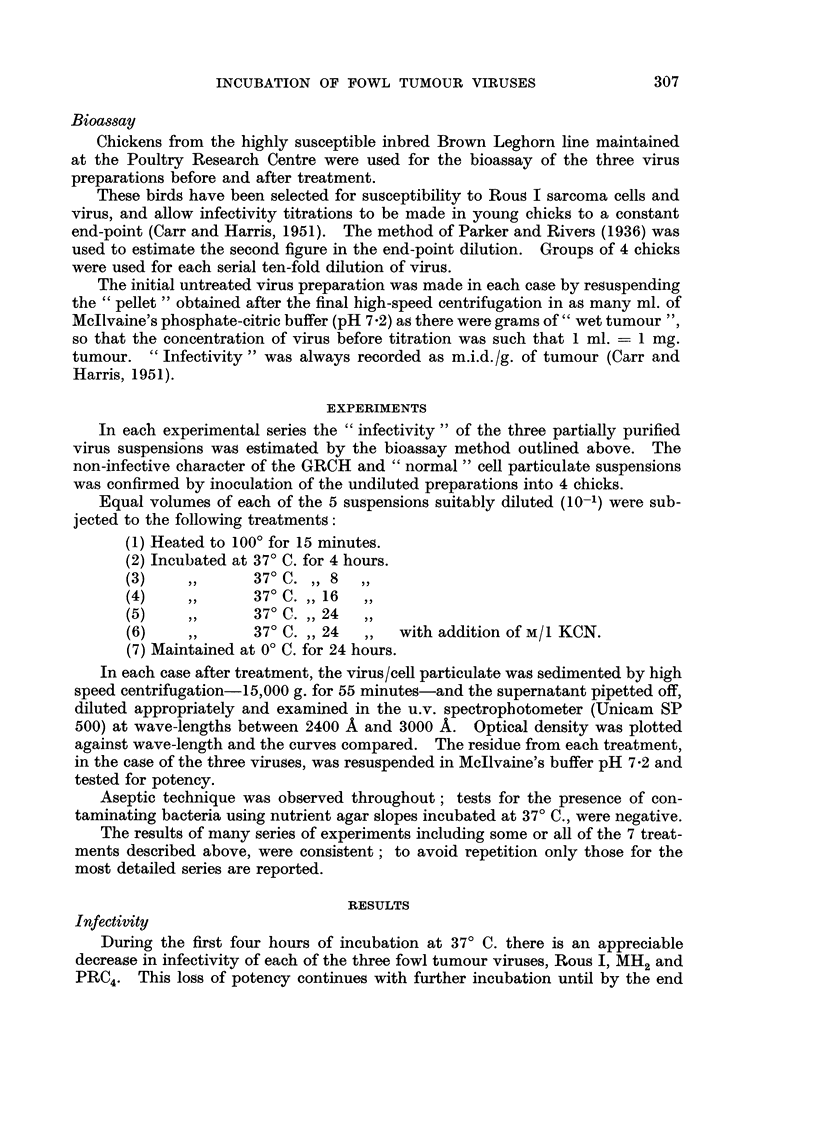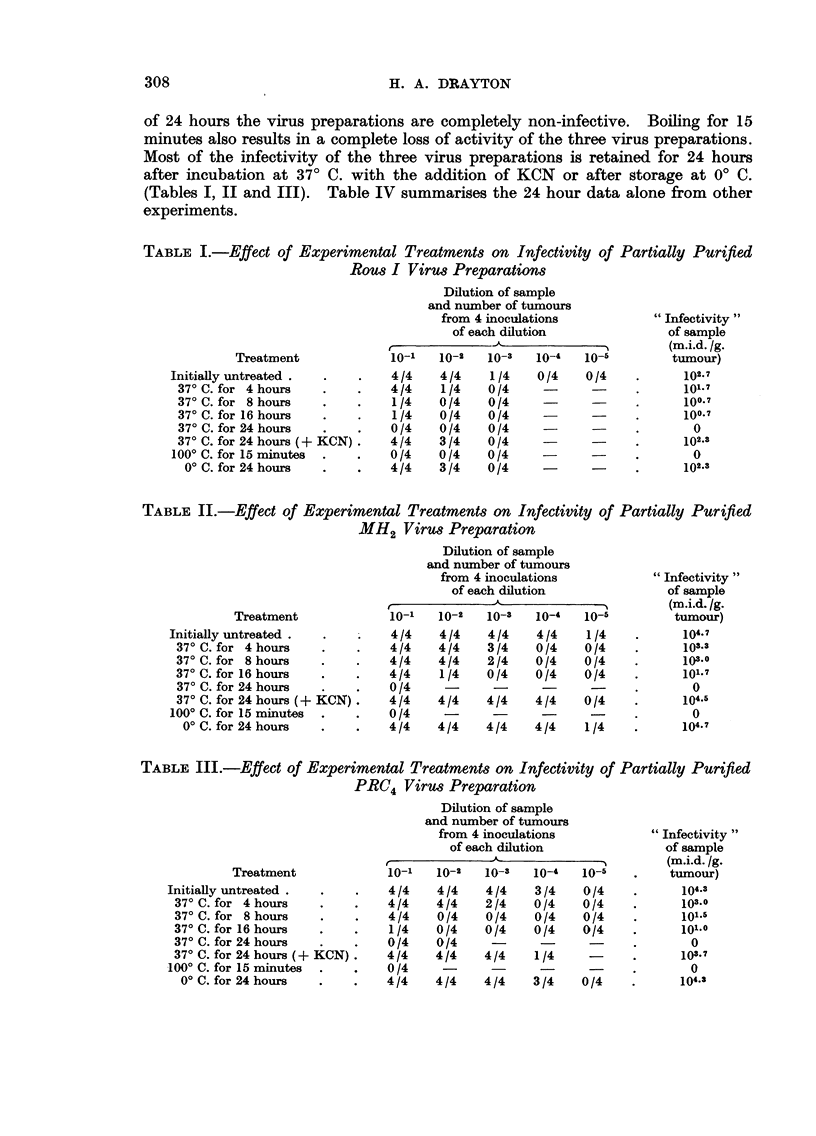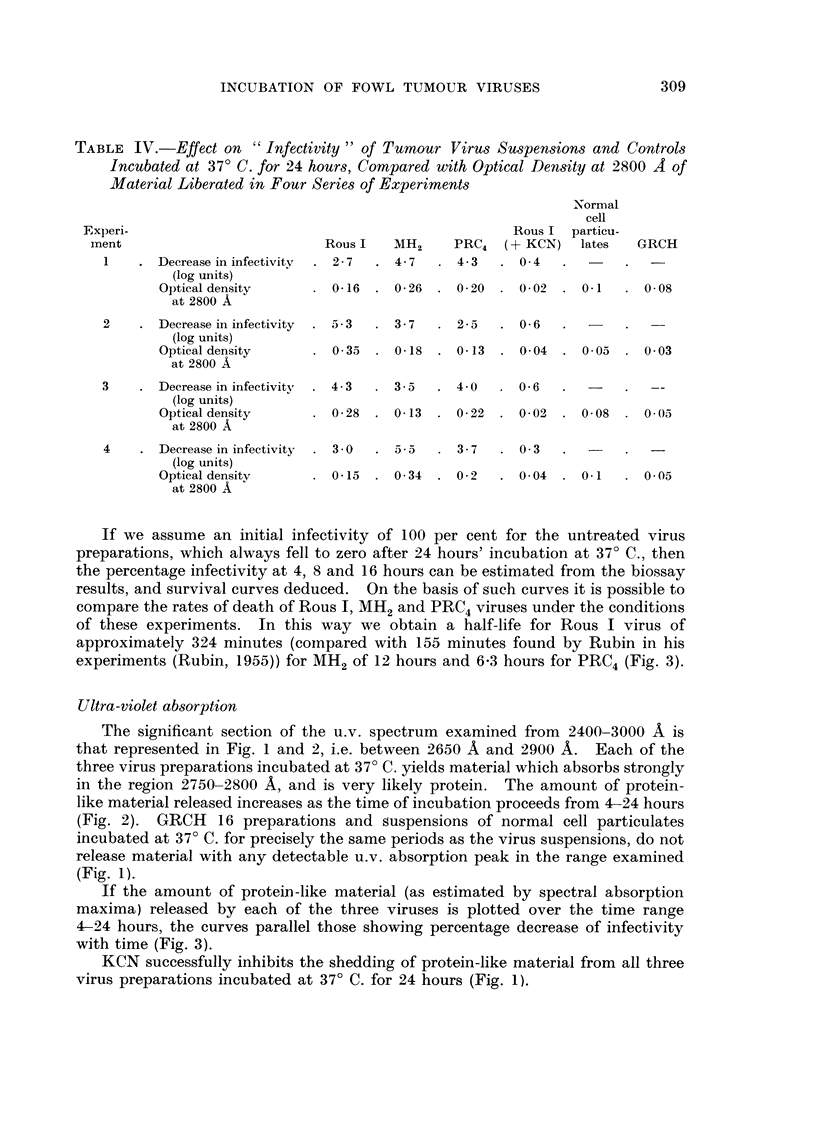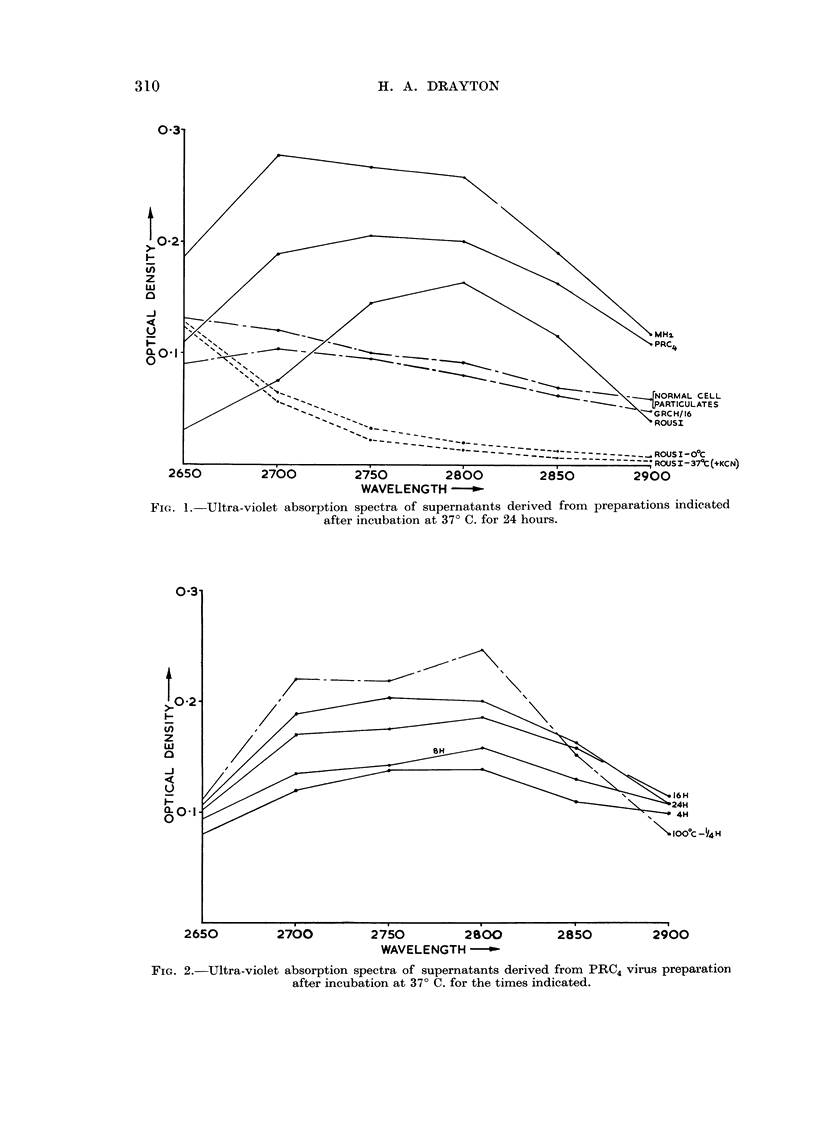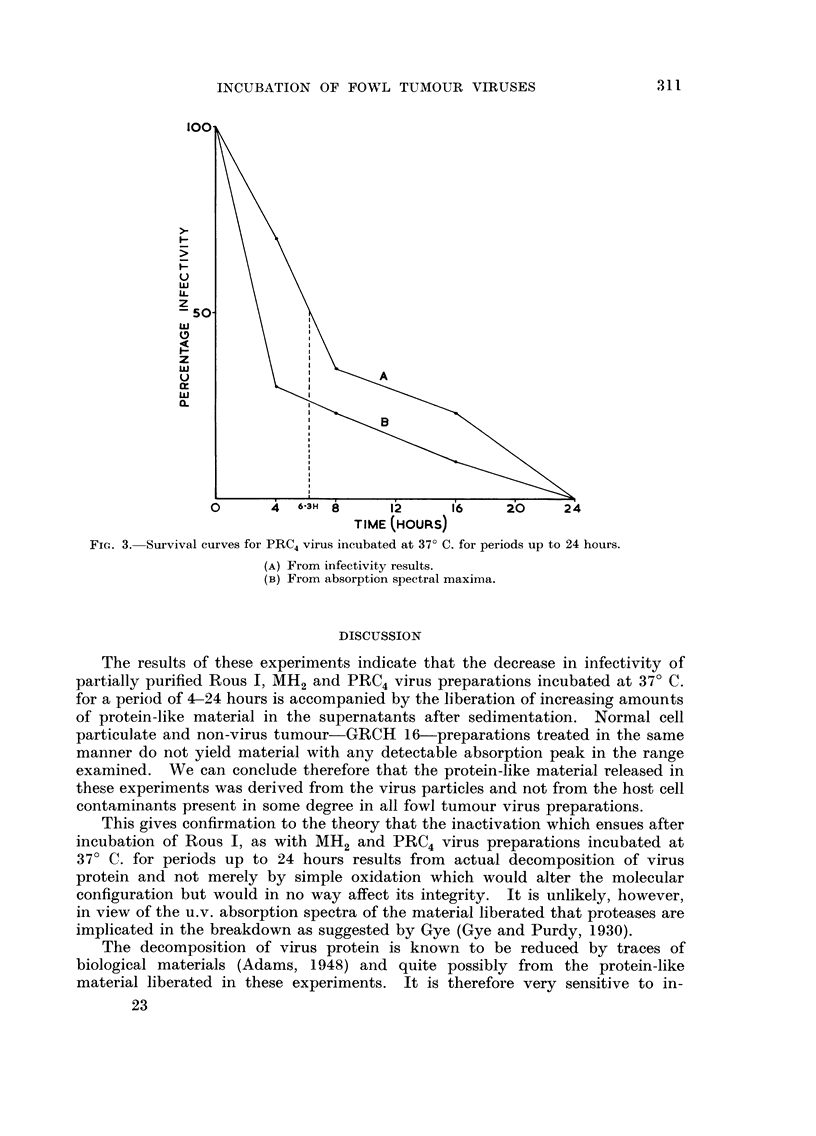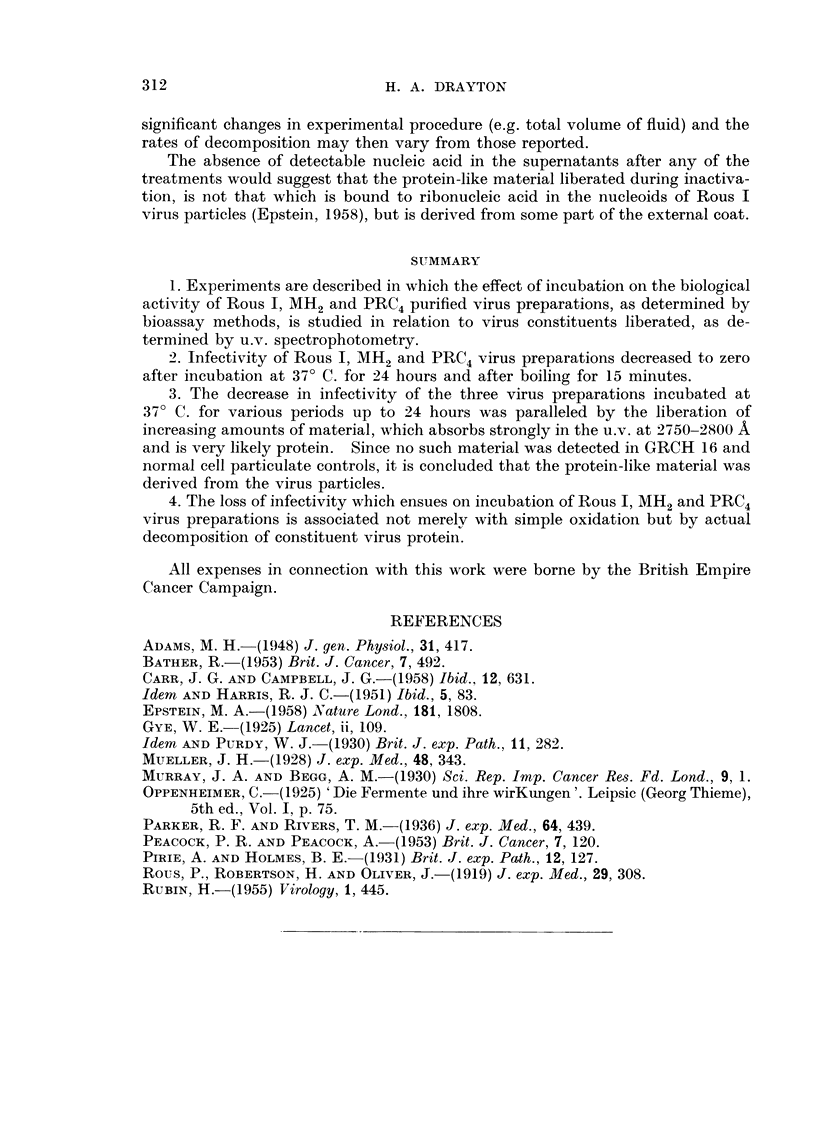# The Effect of Incubation on Three Fowl Tumour Viruses

**DOI:** 10.1038/bjc.1960.36

**Published:** 1960-06

**Authors:** H. A. Drayton


### THE EFFECT OF INCUBATION ON THREE FOWL TUMOUR VIRUSES

H. A. DRAYTON

*From the British Empire Cancer Campaign Unit, Agricultural Research Council Poultry Research Centre, West Mains Road, Edinburgh, 9*

Received for publication April 22, 1960

THE thermolabile character of chicken sarcoma I filtrates has been noted since 1919 (Rous, Robertson and Oliver, 1919). Later, it was found that incubation at 37° C. for 24 hours resulted in a rapid loss of infectivity even in filtrates of high titre and that inactivation was invariably complete after treatment at 55° C. for 15 minutes. Mueller (1928) first mentioned Gye's finding that this inactivation of Rous I filtrates at moderate temperatures could be prevented for periods up to three days by the addition of HCN.

Gye (1925) ascribed this phenomenon of inactivation to the oxidation of a " specific factor ", but later inclined to the view that inactivation was due to " proteolytic ferments in the filtrates acting on a non-living protein specific factor " (Gye and Purdy, 1930). In the light of Oppenheimer's finding that substances like HCN although powerful anti-oxidants, had little effect on proteases (Oppenheimer, 1925), Mueller (1928) adhered to Gye's earlier theory that inactivation was a result of oxidation of a specific component of the virus.

Pirie and Holmes (1931) argued that since incubation at 37° C. even under anaerobic conditions resulted in a loss of potency of Rous I filtrates, atmospheric oxygen need play no part in any oxidation which is assumed to accompany Rous I virus inactivation. They suggested therefore that some oxidiser-catalyst system was closely attached to the virus itself, while admitting that it had not been possible to separate it from the virus nor to identify it chemically.

With the exception of Rous I there is no information on the effect of incubation on the avian tumour viruses. It was thought that a comparative study on the effect of incubation at 37° C. for varying periods of time on the "infectivity " of partially purified Rous I, $MH_2$ (Murray and Begg, 1930), and $PRC_4$ (Carr and Campbell, 1958) virus preparations might be of interest, if an attempt were made to correlate any effect on potency with virus material liberated and detected by u.v. spectrophotometric analysis.

### MATERIALS AND METHODS

### *Preparation of normal cell particulate suspensions and of virus suspensions*

Suspensions of cell particulates from normal fowl tissues (liver and spleen), from a non-filterable fowl sarcoma, GRCH 16 (Peacock and Peacock, 1953) and partially purified virus suspensions from Rous I, $MH_2$ and $PRC_4$ tumours, were prepared by the method originally devised by Carr and Harris (1951) and modified by Bather (1953). In each series of experiments the same initial weight of tissue was used as starting material for the preparation of each of the five suspensions.

*Bioassay*

Chickens from the highly susceptible inbred Brown Leghorn line maintained at the Poultry Research Centre were used for the bioassay of the three virus preparations before and after treatment.

These birds have been selected for susceptibility to Rous I sarcoma cells and virus, and allow infectivity titrations to be made in young chicks to a constant end-point (Carr and Harris, 1951). The method of Parker and Rivers (1936) was used to estimate the second figure in the end-point dilution. Groups of 4 chicks were used for each serial ten-fold dilution of virus.

The initial untreated virus preparation was made in each case by resuspending the " pellet " obtained after the final high-speed centrifugation in as many ml. of McIlvaine's phosphate-citric buffer (pH 7·2) as there were grams of " wet tumour ", so that the concentration of virus before titration was such that 1 ml. = 1 mg. tumour. " Infectivity " was always recorded as m.i.d./g. of tumour (Carr and Harris, 1951).

EXPERIMENTS

In each experimental series the " infectivity " of the three partially purified virus suspensions was estimated by the bioassay method outlined above. The non-infective character of the GRCH and " normal " cell particulate suspensions was confirmed by inoculation of the undiluted preparations into 4 chicks.

Equal volumes of each of the 5 suspensions suitably diluted ($10^{-1}$) were subjected to the following treatments:

(1) Heated to 100° for 15 minutes.

(2) Incubated at 37° C. for 4 hours.

(3) ,, 37° C. ,, 8 ,,

(4) ,, 37° C. ,, 16 ,,

(5) ,, 37° C. ,, 24 ,,

(6) ,, 37° C. ,, 24 ,, with addition of M/1 KCN.

(7) Maintained at 0° C. for 24 hours.

In each case after treatment, the virus/cell particulate was sedimented by high speed centrifugation—15,000 g. for 55 minutes—and the supernatant pipetted off, diluted appropriately and examined in the u.v. spectrophotometer (Unicam SP 500) at wave-lengths between 2400 Å and 3000 Å. Optical density was plotted against wave-length and the curves compared. The residue from each treatment, in the case of the three viruses, was resuspended in McIlvaine's buffer pH 7·2 and tested for potency.

Aseptic technique was observed throughout ; tests for the presence of contaminating bacteria using nutrient agar slopes incubated at 37° C., were negative.

The results of many series of experiments including some or all of the 7 treatments described above, were consistent ; to avoid repetition only those for the most detailed series are reported.

RESULTS

*Infectivity*

During the first four hours of incubation at 37° C. there is an appreciable decrease in infectivity of each of the three fowl tumour viruses, Rous I, $MH_2$ and $PRC_4$. This loss of potency continues with further incubation until by the end of 24 hours the virus preparations are completely non-infective. Boiling for 15 minutes also results in a complete loss of activity of the three virus preparations. Most of the infectivity of the three virus preparations is retained for 24 hours after incubation at 37° C. with the addition of KCN or after storage at 0° C. (Tables I, II and III). Table IV summarises the 24 hour data alone from other experiments.

TABLE I.—*Effect of Experimental Treatments on Infectivity of Partially Purified Rous I Virus Preparations*

| Treatment | Dilution of sample and number of tumours from 4 inoculations of each dilution | | | | | "Infectivity" of sample (m.i.d./g. tumour) |
|---|---|---|---|---|---|---|
| | $10^{-1}$ | $10^{-2}$ | $10^{-3}$ | $10^{-4}$ | $10^{-5}$ | |
| Initially untreated | 4/4 | 4/4 | 1/4 | 0/4 | 0/4 | $10^{2.7}$ |
| 37° C. for 4 hours | 4/4 | 1/4 | 0/4 | — | — | $10^{1.7}$ |
| 37° C. for 8 hours | 1/4 | 0/4 | 0/4 | — | — | $10^{0.7}$ |
| 37° C. for 16 hours | 1/4 | 0/4 | 0/4 | — | — | $10^{0.7}$ |
| 37° C. for 24 hours | 0/4 | 0/4 | 0/4 | — | — | 0 |
| 37° C. for 24 hours (+ KCN) | 4/4 | 3/4 | 0/4 | — | — | $10^{2.3}$ |
| 100° C. for 15 minutes | 0/4 | 0/4 | 0/4 | — | — | 0 |
| 0° C. for 24 hours | 4/4 | 3/4 | 0/4 | — | — | $10^{2.3}$ |

TABLE II.—*Effect of Experimental Treatments on Infectivity of Partially Purified $MH_2$ Virus Preparation*

| Treatment | Dilution of sample and number of tumours from 4 inoculations of each dilution | | | | | "Infectivity" of sample (m.i.d./g. tumour) |
|---|---|---|---|---|---|---|
| | $10^{-1}$ | $10^{-2}$ | $10^{-3}$ | $10^{-4}$ | $10^{-5}$ | |
| Initially untreated | 4/4 | 4/4 | 4/4 | 4/4 | 1/4 | $10^{4.7}$ |
| 37° C. for 4 hours | 4/4 | 4/4 | 3/4 | 0/4 | 0/4 | $10^{3.3}$ |
| 37° C. for 8 hours | 4/4 | 4/4 | 2/4 | 0/4 | 0/4 | $10^{3.0}$ |
| 37° C. for 16 hours | 4/4 | 1/4 | 0/4 | 0/4 | 0/4 | $10^{1.7}$ |
| 37° C. for 24 hours | 0/4 | — | — | — | — | 0 |
| 37° C. for 24 hours (+ KCN) | 4/4 | 4/4 | 4/4 | 4/4 | 0/4 | $10^{4.5}$ |
| 100° C. for 15 minutes | 0/4 | — | — | — | — | 0 |
| 0° C. for 24 hours | 4/4 | 4/4 | 4/4 | 4/4 | 1/4 | $10^{4.7}$ |

TABLE III.—*Effect of Experimental Treatments on Infectivity of Partially Purified $PRC_4$ Virus Preparation*

| Treatment | Dilution of sample and number of tumours from 4 inoculations of each dilution | | | | | "Infectivity" of sample (m.i.d./g. tumour) |
|---|---|---|---|---|---|---|
| | $10^{-1}$ | $10^{-2}$ | $10^{-3}$ | $10^{-4}$ | $10^{-5}$ | |
| Initially untreated | 4/4 | 4/4 | 4/4 | 3/4 | 0/4 | $10^{4.3}$ |
| 37° C. for 4 hours | 4/4 | 4/4 | 2/4 | 0/4 | 0/4 | $10^{3.0}$ |
| 37° C. for 8 hours | 4/4 | 0/4 | 0/4 | 0/4 | 0/4 | $10^{1.5}$ |
| 37° C. for 16 hours | 1/4 | 0/4 | 0/4 | 0/4 | 0/4 | $10^{1.0}$ |
| 37° C. for 24 hours | 0/4 | 0/4 | — | — | — | 0 |
| 37° C. for 24 hours (+ KCN) | 4/4 | 4/4 | 4/4 | 1/4 | — | $10^{3.7}$ |
| 100° C. for 15 minutes | 0/4 | — | — | — | — | 0 |
| 0° C. for 24 hours | 4/4 | 4/4 | 4/4 | 3/4 | 0/4 | $10^{4.3}$ |

TABLE IV.—*Effect on "Infectivity" of Tumour Virus Suspensions and Controls Incubated at 37° C. for 24 hours, Compared with Optical Density at 2800 Å of Material Liberated in Four Series of Experiments*

| Experiment | | Rous I | $MH_2$ | $PRC_4$ | Rous I (+ KCN) | Normal cell particulates | GRCH |
|---|---|---|---|---|---|---|---|
| 1 | Decrease in infectivity (log units) | 2·7 | 4·7 | 4·3 | 0·4 | — | — |
| | Optical density at 2800 Å | 0·16 | 0·26 | 0·20 | 0·02 | 0·1 | 0·08 |
| 2 | Decrease in infectivity (log units) | 5·3 | 3·7 | 2·5 | 0·6 | — | — |
| | Optical density at 2800 Å | 0·35 | 0·18 | 0·13 | 0·04 | 0·05 | 0·03 |
| 3 | Decrease in infectivity (log units) | 4·3 | 3·5 | 4·0 | 0·6 | — | — |
| | Optical density at 2800 Å | 0·28 | 0·13 | 0·22 | 0·02 | 0·08 | 0·05 |
| 4 | Decrease in infectivity (log units) | 3·0 | 5·5 | 3·7 | 0·3 | — | — |
| | Optical density at 2800 Å | 0·15 | 0·34 | 0·2 | 0·04 | 0·1 | 0·05 |

If we assume an initial infectivity of 100 per cent for the untreated virus preparations, which always fell to zero after 24 hours' incubation at 37° C., then the percentage infectivity at 4, 8 and 16 hours can be estimated from the biossay results, and survival curves deduced. On the basis of such curves it is possible to compare the rates of death of Rous I, $MH_2$ and $PRC_4$ viruses under the conditions of these experiments. In this way we obtain a half-life for Rous I virus of approximately 324 minutes (compared with 155 minutes found by Rubin in his experiments (Rubin, 1955)) for $MH_2$ of 12 hours and 6·3 hours for $PRC_4$ (Fig. 3).

*Ultra-violet absorption*

The significant section of the u.v. spectrum examined from 2400–3000 Å is that represented in Fig. 1 and 2, i.e. between 2650 Å and 2900 Å. Each of the three virus preparations incubated at 37° C. yields material which absorbs strongly in the region 2750–2800 Å, and is very likely protein. The amount of protein-like material released increases as the time of incubation proceeds from 4–24 hours (Fig. 2). GRCH 16 preparations and suspensions of normal cell particulates incubated at 37° C. for precisely the same periods as the virus suspensions, do not release material with any detectable u.v. absorption peak in the range examined (Fig. 1).

If the amount of protein-like material (as estimated by spectral absorption maxima) released by each of the three viruses is plotted over the time range 4–24 hours, the curves parallel those showing percentage decrease of infectivity with time (Fig. 3).

KCN successfully inhibits the shedding of protein-like material from all three virus preparations incubated at 37° C. for 24 hours (Fig. 1).

FIG. 1.—Ultra-violet absorption spectra of supernatants derived from preparations indicated after incubation at 37° C. for 24 hours.

FIG. 2.—Ultra-violet absorption spectra of supernatants derived from $PRC_4$ virus preparation after incubation at 37° C. for the times indicated.

FIG. 3.—Survival curves for $PRC_4$ virus incubated at 37° C. for periods up to 24 hours.

(A) From infectivity results.
(B) From absorption spectral maxima.

### DISCUSSION

The results of these experiments indicate that the decrease in infectivity of partially purified Rous I, $MH_2$ and $PRC_4$ virus preparations incubated at 37° C. for a period of 4–24 hours is accompanied by the liberation of increasing amounts of protein-like material in the supernatants after sedimentation. Normal cell particulate and non-virus tumour—GRCH 16—preparations treated in the same manner do not yield material with any detectable absorption peak in the range examined. We can conclude therefore that the protein-like material released in these experiments was derived from the virus particles and not from the host cell contaminants present in some degree in all fowl tumour virus preparations.

This gives confirmation to the theory that the inactivation which ensues after incubation of Rous I, as with $MH_2$ and $PRC_4$ virus preparations incubated at 37° C. for periods up to 24 hours results from actual decomposition of virus protein and not merely by simple oxidation which would alter the molecular configuration but would in no way affect its integrity. It is unlikely, however, in view of the u.v. absorption spectra of the material liberated that proteases are implicated in the breakdown as suggested by Gye (Gye and Purdy, 1930).

The decomposition of virus protein is known to be reduced by traces of biological materials (Adams, 1948) and quite possibly from the protein-like material liberated in these experiments. It is therefore very sensitive to insignificant changes in experimental procedure (e.g. total volume of fluid) and the rates of decomposition may then vary from those reported.

The absence of detectable nucleic acid in the supernatants after any of the treatments would suggest that the protein-like material liberated during inactivation, is not that which is bound to ribonucleic acid in the nucleoids of Rous I virus particles (Epstein, 1958), but is derived from some part of the external coat.

### SUMMARY

1. Experiments are described in which the effect of incubation on the biological activity of Rous I, $MH_2$ and $PRC_4$ purified virus preparations, as determined by bioassay methods, is studied in relation to virus constituents liberated, as determined by u.v. spectrophotometry.

2. Infectivity of Rous I, $MH_2$ and $PRC_4$ virus preparations decreased to zero after incubation at 37° C. for 24 hours and after boiling for 15 minutes.

3. The decrease in infectivity of the three virus preparations incubated at 37° C. for various periods up to 24 hours was paralleled by the liberation of increasing amounts of material, which absorbs strongly in the u.v. at 2750–2800 Å and is very likely protein. Since no such material was detected in GRCH 16 and normal cell particulate controls, it is concluded that the protein-like material was derived from the virus particles.

4. The loss of infectivity which ensues on incubation of Rous I, $MH_2$ and $PRC_4$ virus preparations is associated not merely with simple oxidation but by actual decomposition of constituent virus protein.

All expenses in connection with this work were borne by the British Empire Cancer Campaign.

### REFERENCES

Adams, M. H.—(1948) *J. gen. Physiol.*, **31**, 417.
Bather, R.—(1953) *Brit. J. Cancer*, **7**, 492.
Carr, J. G. and Campbell, J. G.—(1958) *Ibid.*, **12**, 631.
*Idem* and Harris, R. J. C.—(1951) *Ibid.*, **5**, 83.
Epstein, M. A.—(1958) *Nature Lond.*, **181**, 1808.
Gye, W. E.—(1925) *Lancet*, ii, 109.
*Idem* and Purdy, W. J.—(1930) *Brit. J. exp. Path.*, **11**, 282.
Mueller, J. H.—(1928) *J. exp. Med.*, **48**, 343.
Murray, J. A. and Begg, A. M.—(1930) *Sci. Rep. Imp. Cancer Res. Fd. Lond.*, **9**, 1.
Oppenheimer, C.—(1925) 'Die Fermente und ihre wirKungen'. Leipsic (Georg Thieme), 5th ed., Vol. I, p. 75.
Parker, R. F. and Rivers, T. M.—(1936) *J. exp. Med.*, **64**, 439.
Peacock, P. R. and Peacock, A.—(1953) *Brit. J. Cancer*, **7**, 120.
Pirie, A. and Holmes, B. E.—(1931) *Brit. J. exp. Path.*, **12**, 127.
Rous, P., Robertson, H. and Oliver, J.—(1919) *J. exp. Med.*, **29**, 308.
Rubin, H.—(1955) *Virology*, **1**, 445.